# Patient and clinical characteristics associated with gout flares in an integrated healthcare system

**DOI:** 10.1007/s00296-015-3284-3

**Published:** 2015-05-20

**Authors:** Nazia Rashid, Gerald D. Levy, Yi-Lin Wu, Chengyi Zheng, River Koblick, T. Craig Cheetham

**Affiliations:** Drug Information Services, Kaiser Permanente SCAL Region, 12254 Bellflower Blvd, Room 106, 1st Floor, Downey, CA 90242 USA; Southern California Permanente Medical Group, Kaiser Permanente Southern California, Downey, CA USA; Department of Research and Evaluation, Kaiser Permanente Southern California, Pasadena, CA USA

**Keywords:** Gout, Gout flares, Urate-lowering therapy, Adherence, Serum uric acid goal

## Abstract

Gout flares have been challenging to identify in retrospective databases due to gout flares not being well documented by diagnosis codes, making it difficult to conduct accurate database studies. Previous studies have used different algorithms, and in this study, we used a computer-based method to identify gout flares. The objectives of this study were to identify gout flares in gout patients newly initiated on urate-lowering therapy and evaluate factors associated with a patient experiencing gout flares after starting drug treatment. This was a retrospective cohort study identifying gout patients newly initiated on a urate-lowering therapy (ULT) during the study time period of January 1, 2007–December 31, 2010. The index date was the first dispensed ULT prescription during the study time period. Patients had to be ≥18 years of age on index date, have no history of prior ULT prescription during 12 months before index date, and were required to have 12 months of continuous membership with drug benefit during pre-/post-index. Electronic chart notes were reviewed to identify gout flares; these reviews helped create a validated computer-based method to further identify patients with gout flares and were categorized into 0 gout flares, 1–2 gout flares, and ≥3 gout flares during the 12 months post-index period. Multivariable logistic regression was used to examine patient and clinical factors associated with gout flares during the 12-month follow-up period. There were 8905 patients identified as the final cohort and 68 % of these patients had one or more gout flares during the 12-month follow-up: 2797 patients (31 %) had 0 gout flares, 4836 (54 %) had 1–2 gout flares, and 1272 patients (14 %) had ≥3 gout flares. Using a multivariate regression analyses, factors independently associated with 1–2 gout flares and ≥3 gout flares versus no gout flares were similar, however, with slight differences, such as younger patients were more likely to have 1–2 gout flares and patients ≥65 years of age had ≥3 gout flares. Factors such as male gender, not attaining sUA goal, having ≥3 comorbidities, diuretics use, no changes in initial ULT dose, and not adhering to ULT all were associated with gout flares versus no gout flares. Using a new method to identify gout flares, we had the opportunity to compare our findings with the previous studies. Our study findings echo other previous studies where older patients, male, diuretics, having a greater number of comorbidities, and non-adherence are more likely to have more gout flares during the first year of newly initiating ULT. There is an unmet need for patients with gout to be educated and managed more closely, especially during the first year.

## Introduction

Acute gout flare is the most common manifestation of gout and has been described as an acute inflammatory reaction with red, swollen, and painful joints [1, 2]. Gout and any of its clinical presentation, such as flares, tophi, and joint damage, are due to urate crystal deposits in joints and tissues. Previous studies have shown that maintaining serum uric acid (sUA) at target levels of <6 mg/dl and adhering to a urate-lowering therapy (ULT) helps to reduce the frequency of gout flares [3–8]. The goal of gout flare treatment is pain relief through the reduction in inflammation and reduction in crystal dissolution [9]. Current treatments for gout flares include the use of nonsteroidal anti-inflammatory medications (NSAIDs), colchicine, and corticosteroids [9]. Gout flares are a common finding in gout patients, and it is important to identify them in clinical trials and observational studies so there is a better understanding on how to manage these patients appropriately [10]. Patients with gout flares have greater healthcare resource utilization, decreased quality of life, and loss of work productivity, leading to an increased economic burden for the patient as well as the healthcare system [6, 11–16]. Thus, it is important to learn how to better manage these patients, to decrease the economic burden, and to help reduce the frequency of gout flares to improve their quality of life.

Gout flares can be challenging to identify in clinical databases due to gout flares not being well documented by diagnosis codes, thus making it difficult to conduct accurate retrospective studies. Previous studies have used different algorithms consisting of diagnosis codes, radiology reports, pharmacy, and medical claims to identify gout flares [4, 5, 13–15, 17, 18]. However, these clinical surrogates have not been validated, which may have underestimated or overestimated the rates of gout flares, have errors of sensitivity such as failure to identify true gout flares, or have errors in specificity such as identification of subjects who did not experience gout flares [4, 5, 13–15, 17–20]. The lack of standardized gout flare definition for observational studies demonstrates the intrinsic difficulty of flare identification [19]. Therefore, we created a computer-based method to automatically identify gout flares using natural language processing (NLP) and machine learning (ML) from electronic clinical notes [20]. Using this method, we had the opportunity to compare our findings with the algorithms used by previous studies. The objectives of this study were to (1) identify the number of gout flares in gout patients who were newly initiated on a ULT using a validated computer-based method and (2) identify patient and clinical factors associated with patients having gout flares compared with those who did not have gout flares during their first year on ULT.

## Materials and methods

### Study setting and dataset

Kaiser Permanente Southern California (KPSC) provides integrated, comprehensive medical services to 3.6 million members through its own facilities, which includes 14 hospitals, 202 outpatient facilities, and a centralized laboratory. Every member receives a unique medical record number that they keep for life. This allows the member to be linked to various clinical and administrative databases including member enrollment and benefits, inpatient and outpatient visits, laboratory test results, and drug dispensing. All aspects of care and interactions with the healthcare delivery system are identified in a comprehensive electronic medical record (EMR) system. In addition, care delivered outside KPSC is identified by a claims system. The KPSC membership represents 15 % of the population in the Southern California region and closely mirrors the Southern California population; it is racially diverse and includes the entire socioeconomic spectrum [21, 22]. The institutional review board for KPSC approved this study.

### Design and study population

**Fig. 1:**
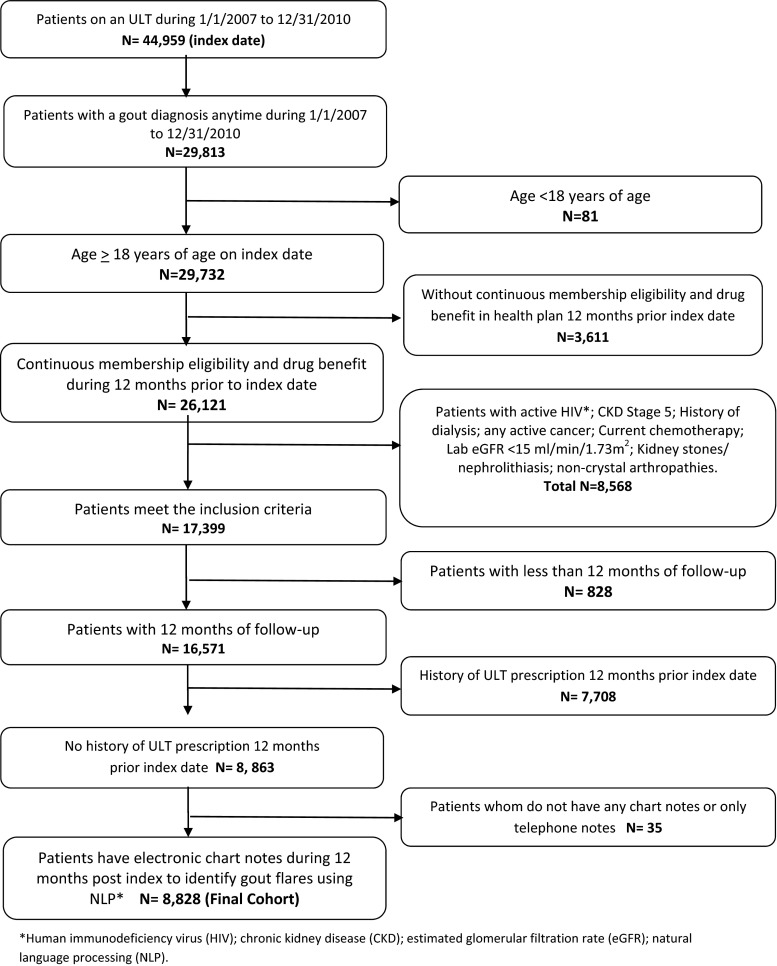
Sample selection flowchart

A retrospective cohort database analysis was conducted during the study time period of January 1, 2007–December 31, 2010. Patients were included if they received a prescription for a ULT (allopurinol, febuxostat, or probenecid) during the study time period; the index date was defined as the patient’s first ULT prescription identified during the study time period. Patients had to be ≥18 years of age at time of index date and were required to have at least 12 months of KPSC membership eligibility including drug benefits prior to their index date, index date, and 12 months post-index. Enrollment gaps of ≤30 days were considered continuous enrollment. Eligible patients were required to have two outpatient gout diagnoses [International Classification of Diseases, Ninth Revision, Clinical Modification (ICD-9-CM) code 274.xx] ≥30 days apart or one inpatient gout diagnosis code in any position anytime during the study time period. We identified patients newly initiating an ULT prescription if they had no ULT prescription during the 12 months prior to their index date. Patients were excluded if they had history of human immunodeficiency virus (HIV), a diagnosis code for chronic kidney disease (CKD) stage 5 or an estimated glomerular filtration rate (GFR) <15 ml/min/1.73 m^2^, history of dialysis, active cancer, current chemotherapy, or kidney stones/nephrolithiasis (Fig. 1). Each exclusion criteria was applied to limit the patient population to those whose primary indication for ULT was gout. All patients had to have chart notes available electronically; patients with only telephone notes, nursing notes, or no chart notes were excluded (Fig. 1).

### Identifying gout flares

In previous studies [4, 5, 13–15, 17, 18], gout flares were identified using ICD-9-CM codes, the use of ULT, the use of symptomatic gout flare medications, and healthcare resource utilization. In this study, we used a validated computer-based method to identify gout flares from free-text clinical notes; the published data and study findings are available elsewhere [20]. As an overview, a search system (beta version) was first created in which search keywords were applied to chart notes. All chart notes for 100 patients were reviewed, and learning from this beta review was implemented to improve the algorithm for the second step, which was defined as the training dataset. The training dataset was created such that a different 100 patients were selected and chart notes were reviewed. Lastly, the refined computer methodology was applied to a final ‘gold standard’ set of progress notes. This method achieved a 82.1 % sensitivity, 91.5 % specificity, 77.9 % positive predictive value (PPV), and 93.4 % negative predictive value (NPV) for identifying gout flares at the note level [20]. Consistent with prior studies [4, 5, 13–15, 17, 18], gout flares and their attendant care were assumed to last for a minimum of 30 days. Two flares within one 30-day period were counted as one flare. Once gout flares were identified, we categorized patients into the following groups during the 12 months of follow-up: 0 gout flares, 1–2 gout flares, and ≥3 gout flares.

### Covariates and measures

Baseline characteristics (during the 12 months prior to the index date and index date) included age, sex, race, comorbid conditions, anti-inflammatory plus other concomitant medication use, initial dose of ULT, sUA levels, renal function, and prescriber specialty. Comorbid conditions included alcohol use, cardiovascular disease, diabetes, hypertension, other arthritic conditions, and obesity. Baseline GFR levels for renal function and baseline sUA levels were measured up to 12 months prior to the index date, and if patients had multiple baseline sUA levels available, the measurement obtained most proximate to the index date was used. Prescription anti-inflammatory medications [nonsteroidals (NSAIDS), colchicine, or corticosteroids] and concomitant medications (antihypertensives, diuretics, anti-hyperlipidemics, anti-diabetics) were all identified from electronic dispensing records. The initial dose of ULT was calculated using pharmacy data which included quantity number, strength, day’s supply, and directions from the dispensed prescription. Prescriber specialty was determined at the time of the index dispensed ULT prescription and was categorized as primary care, rheumatologist, or other.

### Outcomes

Outcomes of interest were identified during post-index for each of the gout flare groups. Outcomes included changes in ULT therapy from baseline such as dose escalation, dose equal, and dose decrease; adherence to ULT being measured using proportion of days covered (PDC) method; utilization of anti-inflammatory therapies during post-index; and sUA goal attainment were identified during post-index. The sUA levels were evaluated for patients that had a baseline and follow-up level. Patients were considered to have had ULT dose escalation if the final observed daily dose was greater than the index dose. Conversely, patients were considered to have had a dose decrease if the final daily ULT dose was less than the initial dose. If there was no change in the ULT dose, then it remained dose equal. PDC was calculated as the number of days with ULT drug dispensed divided by the number of days in the specified time interval (365 days). We evaluated the PDC within the first 12 months of initiating ULT. The PDC was dichotomized <80 % considered as non-adherent and ≥80 % considered adherent. Attainment of sUA goal was defined as the last follow-up sUA obtained after the index date with a value <6.0 mg/dl.

### Statistical analyses

Unadjusted descriptive statistics summarized patient characteristics of the study population where patients with 0 gout flares were compared to patients with 1–2 gout flares and patients with 0 gout flares were compared to patients with ≥3 gout flares. Differences between these patients groups were tested using two-sided *t* test for continuous variables and the Chi-squared statistic for categorical variables. Multivariable logistic regression models were used to evaluate factors associated with patients having 1–2 gout flares versus patients with 0 gout flares and patients with ≥3 gout flares versus patients with 0 gout flares. Factors including age, sex, race, severity of comorbid conditions, diuretic use, prescriber specialty, and anti-inflammatory medications were controlled for in the models. All data were analyzed using SAS version 9.2 (SAS Institute, Cary, NC, USA). *p* values <0.05 were considered to be statistically significant.

## Results

There were 8828 gout patients identified as newly initiated on a ULT, using our selection criteria (Fig. 1); 68 % of these patients had one or more gout flares during the 12-month follow-up. With the application of our gout flare identification method, 2780 patients (32 %) had 0 gout flares, 4798 (54 %) had 1–2 gout flares, and 1250 patients (14 %) had ≥3 gout flares during their 12 months post-index. Table 1 summarizes baseline characteristics for these patients. Patients were predominantly male and Caucasian, and the most common comorbidities among the three groups were hypertension, dyslipidemia, and diabetes. Patients with ≥3 gout flares had a higher percentage of comorbidities versus the other groups, and 47.3 % of them were ≥65 years of age. There were 5826 patients (65 %) with both a baseline and follow-up sUA level. The mean baseline sUA level was lowest in the 0 gout flare group (8.73 ± 1.5 mg/dl) and highest in the ≥3 gout flare group (9.26 ± 1.8 mg/dl). The mean baseline eGFR was lowest in patients ≥3 gout flares (65.01 ± 18.09 ml/min). Patients with ≥3 gout flares had more use of antihypertensives and diuretics versus patients with gout flares. The initial ULT was most commonly prescribed by primary care physicians; however, patients with 0 gout flares had higher percentage of rheumatologists prescribing their initial ULT. The mean initial baseline dose of ULT was higher in the patients with 0 gout flares versus those with 1–2 or ≥3 gout flares at baseline (Table 2).

**Table 1:** Baseline characteristics of study population categorized by gout flares

Patient and clinical characteristics	Total patients *N* = 8828		
	No gout flares	1–2 gout flares	≥3 gout flares
	*N* = 2780 (32 %)	*N* = 4798 (54 %)	*N* = 1250 (14 %)
Male *n*, (%)	2253 (81.0 %)	3801 (79.2 %)	991 (79.2 %)
Age (years) categories, *n* (%)			
<55	953 (34.2 %)	1970 (41.0 %)*	381 (30.5 %)*
55–64	792 (28.4 %)	1123 (28.5 %)*	277 (22.2 %)*
≥65	1035 (37.2 %)	1705 (35.5 %)	592 (47.3 %)*
Race *n*, (%)*			
Caucasian	1187 (42.7 %)	1972 (41.1 %)	501 (40.1 %)
African-American	392 (14.1 %)	747 (15.6 %)	229 (18.3 %)*
Hispanic	536 (19.3 %)	970 (20.2 %)	268 (21.4 %)
Asian/Pacific Islander	651 (23.4 %)	1076 (22.4 %)	240 (19.2 %)
Other	14 (0.5 %)	33 (0.7 %)	12 (0.9 %)
Laboratory data			
sUA *n*, (%)	2032 (72.6 %)	3886 (80.4 %)*	1175 (92.4 %)*
sUA (mg/dl) mean, SD	8.73 ± 1.55	8.93 ± 1.68	9.26 ± 1.81*
eGFR *n*, (%)	2280 (81.5 %)	3905 (80.7 %)	1088 (85.5 %)
eGFR (ml/min/1.72 m^2^), mean, SD	65.01 ± 18.09	64.79 ± 18.28*	60.99 ± 18.96*
Comorbidities *n*, (%)			
Alcohol use	87 (3.1 %)	181 (3.7 %)	64 (5.0 %)*
Diseases of the heart^β^	460 (16.4 %)	835 (17.3 %)	308 (24.2 %)*
Diabetes mellitus	676 (24.2 %)	1016 (21.0 %)	313 (24.6 %)
Dyslipidemia	1641 (58.7 %)	2679 (55.4 %)	716 (56.3 %)
Hypertension	2001 (71.9 %)	3367 (69.6 %)	949 (74.6 %)
Obesity	641 (22.9 %)	1168 (24.2 %)	302 (23.7 %)
Osteoarthritis	469 (16.9 %)	902 (18.8 %)	310 (24.8 %)*
Rheumatoid arthritis	17 (0.6 %)	32 (0.7 %)	16 (1.3 %)*
Anti-inflammatory medication, *n*, (%)			
NSAIDS^a^	1510 (54.3 %)	3199 (66.7 %)*	839 (67.1 %)*
Corticosteroids	454 (16.2 %)	1456 (30.1 %)*	607 (47.7 %)*
Colchicine	924 (33.0 %)	2515 (52.0 %)*	816 (64.2 %)*
Any of the above	2069 (74.4 %)	4377 (91.2 %)*	1192 (95.4 %)*
Concomitant medications *n*, (%)			
Antihypertensives	2091 (74.8 %)	3469 (71.8 %)	970 (76.3 %)
Diuretics	1331 (47.6 %)	2345 (48.5 %)	701 (55.1 %)*
Anti-hyperlipidemics	1391 (49.8 %)	2199 (45.5 %)	590 (46.5 %)
Anti-diabetics	556 (19.9 %)	807 (16.7 %)*	245 (19.3 %)
Initial ULT prescriber specialty *n*, (%)*			
Primary care prescriber^+^	2027 (72.9 %)	4078 (84.9 %)*	1086 (86.9 %)*
Rheumatologist	509 (18.3 %)	241 (5.0 %)*	13 (1.0 %)*
Other	244 (8.8 %)	524 (10.9 %)	151 (12.1 %)

Patients with 1–2 gout flares were compared to no gout flares group, and patients with ≥3 gout flares were compared to no gout flares group

* p value of <0.05 was statistically significant

^β^Diseases of the cardiovascular or blood vessels: heart failure, ischemic heart disease, deep vein thrombosis, cerebrovascular disease, peripheral vascular disease

^+^Primary care prescriber consisted of family medicine and internal medicine

^a^ *NSAIDS* nonsteroidal anti-inflammatory drugs

**Table 2:** Patient outcomes during 12 months post-index

Outcomes	Total patients: *N* = 8828		
	No gout flares	1–2 gout flares	≥3 gout flares
	*N* = 2780	*N* = 4798	*N* = 1250
Adherence to ULT (PDC %)			
Adherent (PDC ≥ 80 %)	2042 (73.5 %)	1861 (38.8 %)*	352 (28.2 %)*
Non-adherent (PDC < 80 %)	738 (26.5 %)	2937 (61.2 %)*	897 (71.8 %)*
ULT treatment information, *n* (%)			
Dose increase	2610 (94.6 %)	791 (16.5 %)*	67 (5.3 %)*
Dose equal	153 (5.5 %)	3927 (81.8 %)*	1146 (91.7 %)*
Dose decrease	17 (0.6 %)	80 (1.7 %)*	37 (3.0 %)*
Laboratory data at end of follow-up			
sUA, *n* (%)	1559 (56.0 %)	3150 (65.1 %)*	1117 (88.0 %)*
sUA levels, mean, SD	5.82 ± 0.73	7.57 ± 1.98*	8.64 ± 1.56*
At goal <6.0 mg/dl) (%)	91.4 %	45.8 %*	21.2 %*
Anti-inflammatory medication during 12 months post-index, *n* (%)			
NSAIDS	1745 (62.8 %)	3611 (75.2 %)*	954 (76.3 %)*
Corticosteroids	652 (23.38 %)	2006 (41.5 %)*	875 (68.9 %)*
Colchicine	1065 (38.08 %)	2994 (61.9 %)*	1036 (81.4 %)*
Any of the above	2288 (82.3)	4658 (97.0 %)*	1242 (99.4 %)*
ULT initial and last doses, mean, SD			
Initial allopurinol dose, mean, SD	202.94 ± 100.54	202.76 ± 120.50	194.18 ± 100.35
Ending allopurinol dose, mean, SD	230.41 ± 109.16	218.80 ± 99.85*	206.53 ± 98.33*
Initial Febuxostat dose, mean, SD	60.00 ± 28.28	50.00 ± 18.52*	35.79 ± 8.38*
Ending Febuxostat dose, mean, SD	53.82 ± 22.73	52.00 ± 19.32	45.57 ± 21.33*
Initial probenecid dose, mean, SD	690.00 ± 362.86	681.82 ± 429.18	590.52 ± 218.14*
Ending probenecid dose, mean, SD	926.88 ± 403.80	789.72 ± 364.12*	752.00 ± 268.74*

Patients with 1–2 gout flares were compared to no gout flares group, and patients with ≥gout flares were compared to no gout flares group

* p value of 0.05 was statistically significant

Table 2 summarizes treatment adherence, ULT dose changes from baseline, sUA goal attainment, and the use of anti-inflammatory medications during follow-up. Patients with 0 gout flares were 74 % adherent to their ULT, while patients with 1–2 or ≥ 3 gout flares were 39 and 28 % adherent, respectively. Majority (95 %) had a ULT increase in dose during follow-up for the 0 gout flares; only 18 and 8 % of patients with 1–2 and ≥3 gout flares had changes in their initial ULT doses during follow-up. The mean sUA level was (5.82 ± 0.7 mg/dl) for patients with 0 gout flares versus (8.64 ± 1.5 mg/dl) for patients with ≥3 gout flares (Table 2). In the 0 gout flares group, 91 % achieved sUA goal compared with 45.8 % in the 1–2 gout flare group and only 21.2 % in the ≥3 gout flares group. There was also higher utilization of any anti-inflammatory medication for patients with flares versus those with no gout flares (Table 2).

Using multivariate logistic regression analysis, we identified patient and clinical factors that were independently associated with patients who were having gout flares versus patients who were not having gout flares (Table 3). Factors associated with 1–2 gout flares and ≥3 gout flares versus no gout flares were similar but more pronounced in those with more flares (≥3 flares). Age ≥ 65, male gender, and having ≥3 comorbidities were associated with increased rate of flares. Patients on diuretics were 19 % more likely to have 1–2 gout flares and 23 % more likely to have ≥3 more gout flares. Other factors such as having index ULT prescribed by primary care, and higher sUA levels were all associated with an increased likelihood of having gout flares.

**Table 3:** Multivariate logistic regression of factors associated with patients with gout flares versus no gout flares during 12-month follow-up in gout patients

Study covariates	Patients with 1–2 flares versus patients with no gout flares^α^	Patients with ≥3 flares versus patients with no gout flares^α^
	OR (95 % CI)	OR (95 % CI)
Male patient (vs. female)	1.53 (1.22, 1.66)	1.95 (1.88, 2.12)*
Patient age categories		
<65 years (reference group)	1.00	1.00
≥65 years	0.94 (0.82, 1.16)	**1.81 (1.16**, **2.52)**
Race		
Caucasian (reference group)	1.00	1.00
African-American	1.02 (0.88, 1.18)	1.20 (0.98, 1.46)
Hispanic	0.96 (0.84, 1.09)	1.06 (0.87, 1.29)
Asian/Pacific Islander	1.03 (0.91, 1.17)	1.14 (0.95, 1.37)
sUA data		
sUA level above 6.0 mg/dl	**1.64 (1.47**, **1.88)**	**3.61 (3.37**, **4.01)**
Comorbidity		
1 comorbidity	**0.68 (0.59**, **0.91)**	**0.79 (0.69**, **0.91)**
2 comorbidities	1.14 (0.96,1.24)	**1.67 (1.31**, **1.87)**
3 or more comorbidities	**1.34 (1.13**, **1.78)**	**1.93 (1.65**, **2.27)**
Other covariates		
Anti-inflammatory	**2.65 (2.21**, **3.19)**	**3.10 (2.51**, **4.02)**
Diuretics	**1.19 (1.05**, **1.34)**	**1.23 (1.01**, **1.57)**
Rheumatologist as initial prescriber	**0.79 (0.69**, **0.91)**	**0.77 (0.66**, **0.89)**

*OR* odds ratio, *CI* Confidence interval

* Highlighted in *bold* means they are statistically significant

^α^Adjusted for sex, age, race, sUA levels, comorbidities, anti-inflammatory medications, diuretic use, and rheumatologist as a prescriber

## Discussion

This is the first study to identify gout flares utilizing a novel validated NLP + ML computer-based algorithm using text searches of clinical notes in EMR. This method provided results with a much higher sensitivity and specificity, when compared to the other database algorithm methods: 82.1 % sensitivity, 91.5 % specificity, 77.9 % positive predictive value (PPV), and 93.4 % negative predictive value (NPV) for identifying gout flares at the note level [20]. Compared with other studies using code-based algorithms to identify flares, the percentage of patients with ≥1 flare is 68 % in our study, compared with 11 % found by Primatesta et al. [25], 35 % by Sarawate et al. [5], 40.9 % by Wu et al. [15], and 45.2 % by Saseen et al. [17]. These differences may be due to the fact that patients often manage their flares at home without entering the healthcare system and generating codes. Providers then document the flares in progress notes at the next scheduled visit; thus, causal references in the notes may not be coded, and within a traditional system, the true incidence of gout flares maybe underreported. The study population along with the definition of gout flare may also be different in our study [4, 5, 13–15, 17, 18, 20].

Gout is associated with many comorbidities, such as obesity, diabetes, renal insufficiency [11, 14, 23–28], and lifestyle-related behaviors such as increase in alcohol intake, consumption of purine rich foods, in particular meats and oily fishes, which may complicate the adequate control of gout. Utilization of diuretics and chemotherapeutic agents is risk factor as well [26–28]. Comorbidities, being of male gender, age, and lifestyle-related behaviors complicate the adequate control of gout [26–28]. Some of these are modifiable, and others are not modifiable [26, 29]. In this study, we found that patients with multiple comorbidities or ≥3 comorbidities had an increase rate in flares [30]. These comorbidities included hypertension, dyslipidemia, cardiovascular disease, and diabetes. The mean baseline eGFR was lowest in patients ≥3 gout flares, showing that renal function was more decreased in this group versus other groups. Patients ≥65 years had a higher incidence of ≥3 gout flares versus in the other groups. This could be contributed to having more comorbidities, being older, and multiple concomitant therapies which may lead to inadequate control of gout; however, there are other studies discussing how age is not a risk factor for gout flares. These maybe contributed to different population, different definition of gout flares, and how a dataset may have been created [26–28, 31, 32].

The relationship between high sUA levels and the recurrent gout flares has been shown in previous studies [3–5]. Consistent with Halpern et al. and Sarawate et al., we found a positive relationship between sUA levels and gout flares. We also found that patients with no gout flares initially had lower sUA levels at baseline versus patients with gout flares [3]. The sUA levels during follow-up showed that patients at goal or having a sUA < 6 mg/dl had no gout flares or less frequent flares versus patients with frequent flares of ≥3 flares. In patients newly initiated on ULT, there have been temporal patterns of gout flares in which we see greater occurrence of gout flares during the first year with ULT use which decrease over time [3–5]. Patients at goal have less likelihood of gout flares, and those with a reduction in sUA levels with continuous ULT are associated with long-term periods of patients being free from gout flares [3, 7, 23, 24]. Poor adherence to ULTs is not successful in keeping sUA levels below goal and increases the incidence of gout flares [3–5, 23, 24]. There could be other factors associated with adherence which could not have been identified, but being non-adherent to ULT medication leads to gout flares and failure to adjust ULT therapy are modifiable causes of gout flare. Patients who had their ULT prescriber by Rheumatologists had less gout flares versus those where the index ULT prescribed by a non-Rheumatologist. Utilization of anti-inflammatory medication during baseline period is positively related to the gout flares in this study; however, we do not know if these specific medications were used for gout flares, prophylaxis, or other reasons. We could not identify if patients took these medications prior to a gout flare since majority of patients may have self-treated their attacks and thus could not calculate a rate. The NLP process identified colchicine use but generally could not distinguish when it was used for prophylaxis versus the use for gout flares. It was difficult to know whether patients were taking this medication as prescribed or consistently versus inconsistently; thus, we could evaluate the duration of use. Our study is consistent with gout management guidelines [9] and other studies that the use of prophylaxis therapy [30] is needed to help control gout flares, and this could also be the possible reason for the increase use of anti-inflammatory medications.

As with any retrospective study, there are some limitations that need to be addressed. One is investigator dependence on the availability and accuracy of the pharmacy and medical records to identify the study population. To help alleviate this concern, we identified patients with gout using two gout diagnoses ≥30 days apart or one inpatient gout diagnosis code, in addition to a prescription for urate-lowering therapy. Another limitation is that the NLP may not be able to identify all patients with acute gouty flares even with the search criteria being established, since gout flares are sometimes self-managed at home or that gout flares are scantly documented. Our validated algorithm with 82.1 % sensitivity and 91.5 % specificity was shown to be superior to traditional methods of identifying gout [20]. Another limitation is that the NLP + ML computer-based method is currently not available in other health plans. However, discussions with other EMR data health plans are in process. Some patients may have filled their medication prescriptions at other non-KPSC pharmacies, and this cannot be identified or patients purchasing their NSAIDs from over the counter for possible gout flares. Also, utilization of NSAIDs could also be used for other conditions, and not just prophylactic use for gout flares. We added the criteria to the selection of the study sample to only include patients who have continuous membership eligibility and drug benefit 12 months pre-index and 12 month post-index. When measuring adherence, we do not know whether the medication was really taken even though it was picked up and recorded in our system. In our health system, we did not identify any patients treated with ACTH for gout flares, and thus, this therapy was not included in the treatment of gout flares. Lastly, patients with CKD stage 5 were excluded, and these risk factors are not applied to this population.

In this study, we used a new and different method to identify gout flares. The validated computer-based method produced findings consistent with other studies, where patients older in age, male, not attaining sUA goal of <6 mg/dl, non-adherence to ULT, diuretic use, and more comorbidities are associated with more gout flares during the first year of newly initiating ULT. Patient education and physician involvement are two keys to reduction in gout flares, especially during the first year where most gout flares occur from initiation of ULT. Patients who follow the recommendations to initiate ULT with a low starting dose or increase the ULT slowly tend to have fewer adverse events [32, 33]. Currently, most of the management of gout occurs in primary care setting and acute gout management is considered suboptimal. In this study, we see that majority of the patients do not have follow-up sUA levels and there is lack of increase to the initial ULT prescription dose. Issues from lack of adherence to suggested treatment, relative contraindications such as hypertension, metabolic syndrome, or chronic kidney disease are among the reasons that gout care and treatment is difficult. These can all lead to continued gout flares. Computer-based methods such as NLP + ML can be used in many healthcare systems where EMR data are available to identify at risk patients, and programs designed to address the modifiable risks can be instituted. More studies related to computer-based methods, evaluating medical resource use, and economic impact of these patients in a managed care integrated system would add to the body of evidence leading to better care for these patients. This study confirms that using a novel computer-based method identifies the importance of modifiable risk factors in the prevention of acute gout flares. ULT dose adjustment and compliance are two factors which can be addressed to lower the frequency and severity of acute gout flares.
